# Microleakage between conventional GIC, RMGIC and glass hybrid bulkfill restorative system to restore class I cavities in primary molars: An *in vitro* study

**DOI:** 10.6026/973206300211279

**Published:** 2025-05-31

**Authors:** Madiha Khan, Satish Maran, Ruba Khan, Ami Rawal, Palak Jain, Ali Khan

**Affiliations:** 1 Department of Pediatric and Preventive Dentistry, Peoples Dental Academy, Bhopal, Madhya Pradesh, India; 2 Department of Conservative Dentistry and Endodontist, Peoples University, Bhopal, Madhya Pradesh, India; 3 Department of Oral and Maxillofacial Pathology, Peoples Dental Academy, Bhopal, Madhya Pradesh, India; 4 Department of Orthodontics and Dentofacial Orthopedics, Rishiraj College Dental Sciences and Research Centre, Bhopal, Madhya Pradesh, India

**Keywords:** Microleakage, bulk filled hybrid restorative system (GIC), resin-modified glass ionomer cements (RMGIC), EQUIA forte

## Abstract

The microleakage of conventional GIC to that of resin modified GIC and bulk filled hybrid restorative system (GIC) to restore class I cavities in primary molars is of interest. Hence, a total of 60 extracted human primary molar teeth collected were divided into three main groups. Class I cavity was prepared in all teeth and restore with conventional GIC, resin modified GIC and bulk filled hybrid restorative system (GIC). Sections were viewed under stereomicroscope and the degree of microleakage was evaluated using specific scoring criteria. Among the three restorative materials, Glass hybrid Bulkfil restorative system GIC can be considered as the best material in the terms of microleakage.

## Background:

Microleakage is the most common cause of restoration failure [1]. In clinical practice micro-leakage leads to marginal breakdown, postoperative hypersensitivity, recurrent caries, pulpal pathologies which are the most common problem in restorative dentistry. The ability of the restorative material to effectively sustain oral conditions depends on the retentive ability of the material to seal the cavity against the entrance of oral fluids and micro-organism is critical for success of any restorative material [2]. There is a wide range of options that can be used as restorative materials to restore carious or non-carious lesions of teeth [3]. As glass ionomer cements require lesser steps and has less setting time, they are the most commonly used restorative materials for primary teeth. It is self-adhesive cement to enamel and dentin and provides caries-protective fluoride release at the margins of restorations, as well as its ability to have the fluoride within the chemical matrix recharged by outside exposure to other fluoride-containing materials [4]. Conventional GICs have the drawbacks like handling and working difficulties, sensitivity to water and brittleness [5]. These shortcomings were improved by adding photosensitive methacrylate resin to glass powder in resin-modified glass ionomer cements (RMGIC) [6]. In RMGIC, adhesion to tooth structure is improved by the formation of hybrid layer from the hydrophilic methacrylate resins besides an ionic bonding like GIC. They produce a catalyst rich air-inhibited layer which can polymerize with the composite, making them advantageous in glass ionomer/composite laminate restorations Application of cavity conditioner before restoration plays an important role in developing effective bond with RMGIC [7]. Another recent advancement in glass ionomer cement is GC EQUIA Forte, which is an innovative restorative system based on a new glass hybrid technology. EQUIA Forte combines a filling component with a protective composite coating while additionally benefiting from a newly developed hybrid filler technology. The resulting restorative offers improved performance in tooth coloured posterior restorations for patients of all generations [8]. Therefore, it is of interest to evaluate and compare microleakage properties of two most commonly used glass ionomers worldwide (conventional glass ionomers - Fuji IX and resin-reinforced glass ionomers - Fuji II LC) with the recently introduced EQUIA Forte (bulk fill glass hybrid restorative system).

## Materials and Methods:

The study subject comprised of 60 extracted human primary molar teeth collected from the patients visiting the Department of Pedodontics and Preventive Dentistry, People's Dental Academy, Bhopal for extraction. The research protocol has been approved by Institutional Human Ethical Committee at People's University, Bhopal. Extracted teeth were stored in distilled water for a maximum of 1 month at 4 C. After surface debridement with hand-scaling instruments and cleansing with a slow-speed hand piece and a brush with pumice, the teeth were randomly divided into 3 groups of 20 teeth each.

## Inclusion criteria:

[1] Exfoliating primary molars with marginal ridge intact

[2] Carious molars with intact marginal ridge

[3] Primary molars with intact marginal ridge which will be extracted for orthodontic purpose (serial extraction)

[4] Over retained primary molars with marginal ridge intact

## Exclusion criteria:

[1] Anterior teeth

[2] Grossly carious primary teeth

[3] Primary teeth not having intact marginal ridge

[4] Primary teeth with complex cavities

[5] Primary teeth having restoration

[6] Primary fractured teeth

[7] Decidous teeth on which pulp therapy has been previously done

## Methodology:

Class I cavity was prepared in all teeth (buccolingual width not exceeding 1/3 intercuspal distance. with 1.5 mm depth).

## Application of restorative materials:

## Application of conventional GIC (Fuji IX Extra) - Group I:

The material was applied as recommended by the manufacturer in group 1 cavities. A plastic instrument was used to remove excess material and then finishing was done.

## Application of RMGIC (Fuji II LC) - Group 2:

The material was applied to the prepared cavities of group2 as recommended by the manufacturer, contoured light cured for 20 sec. and then finishing was done.

## Application of bulk fills glass hybrid restorative system (EQUIA Forte) - Group 3:

Materials were supplied in capsules, mixed using amalgamator (GnatusAmlgamix II) as recommended by the manufacturer and immediately applied in the cavities as a single layer using the Aplicap Applier (3M ESPE dental products, USA). In all cavities, the capsule nozzle started extrusion of restoration at the deepest part of the cavity to avoid incorporation of air bubbles. The material was applied to the prepared cavities of groups group 3, allowed to set freely for 5 minutes, a plastic instrument was used to remove the excess immediately after restoration insertion, finishing was done using super fine finishing diamond thereafter a layer of GC coat was applied to the restoration surface. Light curing of the coat for 20 sec was done using a light-emitting diode (LED) curing unit.

## Thermo-cycling:

After 24 hours storage of teeth in distilled water, thermocycling was done at 5°C and 55°C for 500.

## Immersion in dye:

The marginal sealing of the restored teeth was assessed using dye penetration method. Sealing of the root apices was done by using wax after thermocycling and two coats of nail varnish were coated on teeth 1 mm away from the restoration margin. Teeth were then dipped in 2% methylene blue dye. After 24 hours of immersion at room temperature, teeth were removed, washed under copious amount of water for 10 minute to remove excess dye then left to dry for 6 hours until dye fixation.

## Microleakage assessment:

Each tooth was sectioned vertically in a buccolingual direction through the center of the restoration into two halves using a diamond disc (Brown Alumina Oxide, Henan Tianze Imp and Exp) with a low speed straight hand piece (Allowable max. speed 40,000 rpm weight 48g NSK, Japan) under coolant water spray. One section of each tooth was carefully cleaned with alcohol to remove the cutting debris to be examined under stereomicroscope.

## Stereomicroscope evaluation:

The extent of the leakage was assessed using stereomicroscope at 40X magnification; a photographic record for each specimen was obtained and the degree of dye penetration was scored. The depth of dye penetration was analyzed according to a modified version of 0-3 scale scoring system suggested by Araújo *et al.* [17] (Table 1). Figure 1-4 (see PDF) shows the depth of dye penetration under stereomicroscopic examination.

## Results and observations:

Table 2 summarizes the mean micro-leakage of Conventional GIC, RMGIC & Glass hybrid Bulkfil restorative system score which was found highest among conventional GIC, followed by RMGIC and it was least among group III samples (Glass hybrid Bulkfil restorative system GIC). Mean microleakage score was 2.35±0.813, 1.70±1.129 and 1.05±0.605 among group I, II & III samples. Median score was 3, 2 & 1 among group I, II & III samples. Its range was 0-3.Data distribution was checked by Kolmogorov-Smirnov & Shapiro-Wilk test. It was found that data distribution was skewed so non parametric test Kruskal Wallis was applied. There was statistically highly significant difference found in Microleakage score between conventional GIC, RMGIC & Glass hybrid Bulkfil restorative system GIC (P=0.001). Mean microleakage score was 1.70±1.129 & 1.05±0.605 among group II & III samples. Mann Whiney 'U' test was applied to find significant difference between group II & III. There was statistically significant difference found in mean Microleakage score between RMGIC & Glass hybrid Bulkfil restorative system GIC (P=0.04) (Table 3).

Table 4 reveals comparative evaluation of Microleakage between conventional GIC, RMGIC & Glass hybrid Bulkfil restorative system GIC. Micro leakage was measure on 0-3 scale. Score 0 mean No dye penetration, score 1 is dye penetration Less than the half of cavity depth, score 2 means, More than the half of cavity depth and score 3 means, Till the full cavity depth. Among group I samples, score 3 was found in most of samples *i.e.* 11 samples and score 2 was in 5 samples. Among group II samples, score 2 & 3 was found in 6 samples and score 0 & 1 was found in 4 samples. Among group III samples, score 3 was not seen, score 1 & 2 was found in 13 & 4 samples respectively (Figure 5 - see PDF). The Figure 5 (see PDF) is the graphical representation of the mean score of the groups. There was statistically highly significant difference found in distribution of Microleakage score between conventional GIC, RMGIC & Glass hybrid Bulkfil restorative system GIC (P=0.001).

## Discussion:

In pediatric dentistry, it is crucial to select restorative materials with anti-cariogenic properties, good clinical performance and short operating time, especially while working on children with limited attention span [8]. Restorative dentistry plays an important role in the mechanical, biological and social aspects of dentistry, making aesthetics an appealing feature of restorations in anterior and posterior teeth. One of the main reasons of restoration failure is microleakage, caused by the passage of bacteria, fluids, molecules and/or ions between the cavity walls and the restorative material. Clinically, this can be observed by stains on restoration margins, loss of marginal integrity, recurrent caries at the tooth/ restoration interface and hypersensitivity of restored teeth, in addition to the possible development of pulp pathology [7]. Microleakage evaluation is the method most commonly used for assessing the sealing efficiency of a restorative system Yammazaki *et al.* (2006) [10]. *in vitro* microleakage studies are a valuable means of evaluating clinical restorative materials and techniques on their ability to reduce and perhaps eliminate, microleakage was said by Bertrand *et al.* (2006) [12] Sungurtekin and Oztas, (2010) [11]. This *in vitro* study was carried out to further investigate microleakage of new GICs in primary teeth. This implies that differences may exist between the restorative materials. The mechanical loading leads to a decrease in bonding performance because of fatigue at the adhesive interface. A consolidated view indicates that while various material properties (*e.g.*, thermal expansion, elasticity) seem to play a role in modifying the marginal sealing ability; non material-related factors (*e.g.*, cavity configuration, application technique and curing method) have to be taken into account as well [8]. Castro *et al.* (2002) [13] said long-term maintenance of a marginal seal is extremely important for avoiding or at least decreasing clinical problems such as discoloration of margins due to microleakage and secondary caries. Microleakage is governed by marginal adaptation of the restorative material to the tooth and is influenced by polymerisation shrinkage and coefficient of thermal \expansion. Ideally, filling materials and dental tissues should have identical coefficients of thermal expansion in order to limit leakage at the margins of the restorations [12]. Restorative materials are constantly subjected to thermal challenges in the oral environment. Intraoral temperature changes may be induced by routine eating and breathing [14, 15]. Thermal stresses can be pathogenic in two ways. Firstly, mechanical stresses induced by differential thermal changes can directly induce crack propagation through bonded interfaces [14]. Secondly, the changing gap dimensions are associated with gap volume changes which pump pathogenic oral fluids in and out of the gaps. Thermal cycling is an *in vivo* process often represented in these simulations [15]. To simulate thermal stresses on the tooth restoration interface, microleakage studies usually employ thermocycling of different regimens. In this study, the dynamic environment of the oral cavity was simulated by exposing the restored teeth to thermal changes via thermocycling. Thermal cycles ranging between 100 and 1,000 were used in some studies [11]. In the present study 500 thermal cycles were used. Further, microleakage can be assessed by various methods such as radioisotopes, dyes, air pressure, neutron activation analysis, pH changes and scanning electron microscopy; however, dyes and radioisotopes are most commonly used. In the present study, 2% methylene blue dye was selected as a measure of microleakage because of its low cost, ease of manipulation, convenience and also the low molecular weight of the dye is smaller than bacteria that could detect leakage where bacteria could not penetrate. Dye penetration test was used to assess marginal leakage in this study. It is a widely accepted and generally preferred method because it is readily available, cheap and non-toxic. In addition, the most effective dye for revealing microleakage is Methylene blue 0.5% was used in the study. Which was also used by Sungurtekin and Oztas (2010) [11] in their study and for the total sample, Class I cavities were prepared as they were easy to standardize. Research showed that the smear layer on the prepared cavity walls can affect the bond between GIC and dentin. If the smear layer is not removed, it can act as a weak point leading to cohesive failure during polymerization shrinkage and episodes of thermal expansion and contraction [16]. Several researchers reported improved bond strength when the smear layer was removed using polyacrylic acid before the use of RMGIC [17]. Regardless of the agent used, it must be remembered that GICs form a chelation bond to dentin and enamel and the elimination of the smear layer improves the bonding of the cement to the actual tooth structure. In this study, for the qualitative measurements we measured the degree of microleakage on a 0-3 scale using the same images suggested by Araújo [17]. In the present study we observed that on comparison RMGIC (Fuji II LC) showed less microleakage than conventional GIC (Fuji IX Extra). There was statistically significant difference found in mean microleakage score between Coventional GIC & RMGIC. (P=0.05). Similarly Tjan and Dunn [18] compared the microleakage of RMGIC with Conventional GIC and found that RMGIC showed significantly less leakage than control group. Similar results were observed by studies done by Sindu [19] and Toledano *et al.* [20] Kevin *et al.* [21] in their study. But not all studies have showed such results. Pavaluri *et al.* [22] showed no significant difference in microleakage between conventional GIC and RMGIC.

The marginal seal of restorative materials to the cavity walls depends on two variables. The first refers to material properties such as the type of adhesive, polymerization shrinkage, viscoelasticity and stiffness of material [18]. The second refers to individual treatment conditions which include cavity size, geometry, restorative placement and curing techniques. The clinical implication of microleakage is related to discomfort in conjunction with occlusal forces, which may be attributed to fluid accumulation within the gap and the subsequent fluid movement within the tubules [11]. The results of the present study demonstrate significant differences in microleakage between conventional GIC when compared with RMGIC and EQUIA Forte. The result of this study showed that EQUIA Forte was the only restoration that has the least microleakage followed by Fuji II LC and Fuji IX GP Extra has higher leakage was detected. This was in agreement with another study [23] who found that EQUIA showed less microleakage than RMGICs. This might be due to that the coefficient of thermal expansion of EQUIA is similar to that of adjacent tooth structure, which could be a reason for less microleakage observed in EQUIA as compared to Fuji II LC, while the coefficient of thermal expansion being quite high as compared to tooth structure for the latter done by Amaral *et al.* [24]. While in another study done by Siddiqui *et al.* [25] the EQUIA system showed results very similar to resin modified glass ionomer and this might be due to the fact that it compared other types of RMGICs (Ketac Molar and Photac Fil) with EQUIA system in class I cavities and without thermocycling. The present study revealed that bulk fill hybrid restorative system (EQUIA FORTE) formed a good marginal seal among the all the material tested. A related microleakage study where Class II cavities were restored showed similar mean microleakage scores [26]. EQUIA Forte demonstrated optimal handling features, reduced operating time and favorable marginal seal, leading to the success of this restorative material in the clinical environment. Both these factors are valuable in pediatric dentistry. Accordingly, the long term effects of the restorative materials in oral environments must be monitored. It is worth noting that further studies utilizing advanced micro XCT methods to assess interfacial gap could support the findings of this present work [27].

## Conclusion:

None of the tested GICs was free from microleakage. Cavities filled with conventional GIC (Fuji IX Extra) showed maximum microleakage than RMGIC (FUJI II LC) and then bulk fill hybrid restorative system (EQUIA Forte). In addition, fewer application procedures and reduced treatment time in bulk fill hybrid restorative systems are especially advantageous in pediatric dentistry where younger patients get easily agitated. The combination of these benefits and other results enumerated above may prove valuable to the dental profession.

## Figures and Tables

**Table 1:** Depth of dye penetration scale scoring system [17]

**Scores**	**Scale**
0	No dye penetration
1	Dye penetration along the cavity wall to less than half of the cavity depth
2	Dye penetration along the cavity wall to more than half of the cavity depth but not to the full cavity depth.
3	Dye penetration along the cavity wall to the full cavity depth

**Table 2:** Comparative evaluation of Microleakage between Conventional GIC, RMGIC & Glass hybrid Bulkfil restorative system GIC

**Groups**			**Microleakage Score**			
		**N**	**Mean**	**SD**	**Median**	**Range**
Group I	Conventional GIC	20	2.35	0.813	3	3-Jan
Group II	RMGIC	20	1.7	1.129	2	0-3
Group III	Glass hybrid Bulkfil restorative system GIC	20	1.05	0.605	1	0-2
Kruskal Wallis Test : Chi Square Value			16.838			
Significance 'p' Value			0.001(HS)			

**Table 3:** Comparative evaluation of Microleakage between conventional RMGIC & Glass hybrid Bulkfil restorative system GIC

**Groups**			**Microleakage Score**			
		**N**	**Mean**	**SD**	**Median**	**Range**
Group II	RMGIC	20	1.7	1.129	2	0-3
Group III	Glass hybrid Bulkfil restorative system GIC	20	1.05	0.605	1	0-2
Mann Whitney 'U' Test Value			128			
Significance 'p' Value			0.04(S)			

**Table 4:** Comparative evaluation of Microleakage between conventional GIC, RMGIC & Glass hybrid Bulkfil restorative system GIC

**Microleakage Score**		**Group I**	**Group II**	**Group III**	**Total**
		**Conventional GIC**	**RMGIC**	**Glass hybrid Bulkfil restorative system GIC**	
Score 0	No Dye Penetration	0	4	3	7
Score 1	Less than the half of cavity depth	4	4	13	21
Score 2	More than the half of cavity depth	5	6	4	15
Score 3	Till the full cavity depth	11	6	0	17
TOTAL		20	20	20	60
Chi Square Value		22.534			
Significance 'p' Value		0.001(HS)			
